# Ceragenins and Ceragenin-Based Core-Shell Nanosystems as New Antibacterial Agents against Gram-Negative Rods Causing Nosocomial Infections

**DOI:** 10.3390/pathogens12111346

**Published:** 2023-11-13

**Authors:** Maciej Karasiński, Urszula Wnorowska, Bonita Durnaś, Grzegorz Król, Tamara Daniluk, Karol Skłodowski, Katarzyna Głuszek, Ewelina Piktel, Sławomir Okła, Robert Bucki

**Affiliations:** 1 Department of Medical Microbiology and Nanobiomedical Engineering, Medical University of Białystok, Mickiewicza 2C, 15-222 Bialystok, Poland; maciek.karasinski@gmail.com (M.K.); urszula.wnorowska@umb.edu.pl (U.W.); tamara.daniluk@umb.edu.pl (T.D.); karol.sklodowsky@gmail.com (K.S.); 2 Department of Microbiology and Immunology, Institute of Medical Science, Collegium Medicum, Jan Kochanowski University in Kielce, IX Wieków Kielc 19A, 25-317 Kielce, Poland; bonita.durnas@onkol.kielce.pl (B.D.); g.krol@op.pl (G.K.); katarzyna.gluszek@ujk.edu.pl (K.G.); 3 Independent Laboratory of Nanomedicine, Medical University of Białystok, Mickiewicza 2B, 15-222 Białystok, Poland; ewelina.piktel@umb.edu.pl; 4 Holy Cross Oncology Center of Kielce, Artwińskiego 3, 25-734 Kielce, Poland; slawomir.okla@gmail.com

**Keywords:** healthcare-associated infections, ceragenins, nanosystems, nanoantibiotics, multidrug-resistance Gram-negative bacteria

## Abstract

The growing number of infections caused by multidrug-resistant bacterial strains, limited treatment options, multi-species infections, high toxicity of the antibiotics used, and an increase in treatment costs are major challenges for modern medicine. To remedy this, scientists are looking for new antibiotics and treatment methods that will effectively eradicate bacteria while continually developing different resistance mechanisms. Ceragenins are a new group of antimicrobial agents synthesized based on molecular patterns that define the mechanism of antibacterial action of natural antibacterial peptides and steroid-polyamine conjugates such as squalamine. Since ceragenins have a broad spectrum of antimicrobial activity, with little recorded ability of bacteria to develop a resistance mechanism that can bridge their mechanism of action, there are high hopes that this group of molecules can give rise to a new family of drugs effective against bacteria resistant to currently used antibiotics. Experimental data suggests that core-shell nanosystems, in which ceragenins are presented to bacterial cells on metallic nanoparticles, may increase their antimicrobial potential and reduce their toxicity. However, studies should be conducted, among others, to assess potential long-term cytotoxicity and in vivo studies to confirm their activity and stability in animal models. Here, we summarized the current knowledge on ceragenins and ceragenin-containing nanoantibiotics as potential new tools against emerging Gram-negative rods associated with nosocomial infections.

## 1. Introduction

Nosocomial infections, also known as healthcare-associated infections (HAIs), have emerged as a significant issue, posing a severe threat to patient lives and a challenge to healthcare systems worldwide. HAIs appear no earlier than 48 h following admission to a hospital, although symptoms can also occur subsequent to a patient’s release [1]. According to the World Health Organization [2], the incidence of these occurrences is approximately 7% in underdeveloped countries and 10% in industrialized nations. In the European context, it has been reported that around 3.2 million people are impacted by HAI on an annual basis [3]. The precise global mortality rate associated with healthcare-associated infections remains uncertain; nevertheless, many studies indicate a 30-day mortality rate of approximately 10% among patients who have contracted HAI [4,5]. The incidence of excess mortality resulting from HAI appears to be more pronounced among patients who are critically ill, even when considering admission prognostic variables and severity scores. In the USA, in the context of acute care hospitals, it has been estimated that the annual expenses associated with the five primary forms of HAI amount to around $9.8 billion, alone among the adult inpatient populations [6]. There are a number of HAI, but among the most common are central line-associated bloodstream infections (CLABSI), catheter-associated urinary tract infections (CAUTI), skin and soft tissue infections (SSTI), surgical site infections (SSI), ventilator-associated pneumonia (VAP), hospital-acquired pneumonia (HAP), and *Clostridioides difficile* infection (CDI), with bacteria causing about 90% of HAI [7] (Table 1).

An alarming effect of HAI is the emergence and spread of antibiotic-resistant bacteria, also known as "superbugs." In healthcare contexts, the misuse and overuse of antibiotics contribute to the emergence of resistance, rendering common treatments ineffective and complicating infection management. Gram-negative bacteria, including those belonging to the *Enterobacterales* order, and non-glucose-fermenting bacteria, such as *Pseudomonas* and *Acinetobacter*, stand out as two of the most dangerous pathogens that cause HAIs. Due to high bacterial plasticity, bacteria have adapted to an environment rich in overused/misused antibiotics, resulting in Gram-negative bacteria infections in hospitalized patients that are incurable with conventional antibiotics and pandemic-like [26,27].

The limitations and challenges associated with HAIs encompass several key aspects, including antibiotic resistance, biofilm formation, diagnostic difficulties, treatment complexities, and the importance of preventive measures. One notable instance is the occurrence of biofilm formation on medical devices, which poses challenges to effectively eradicating bacteria. Consequently, this issue contributes to increased expenses related to longer hospitalization, surgical procedures, and the protracted use of antimicrobial treatments. It is of utmost importance for healthcare practitioners and academics to acknowledge and tackle the limits and obstacles that are inherent in the context of HAIs. The effective mitigation of these illnesses necessitates a comprehensive approach that encompasses innovative therapeutic methodologies, stringent infection control measures, and a concerted emphasis on limiting the dissemination of pathogens inside healthcare environments. The comprehension and alleviation of these challenges will ultimately result in enhanced patient outcomes and a decrease in the prevalence of HAIs.

The purpose of this review is to shed light on the alarming prevalence of HAIs, with a particular emphasis on the worrisome emergence of Gram-negative rods as a formidable foe within healthcare facilities. By analyzing the epidemiology, underlying mechanisms, and clinical implications, this review aims to contribute to the collective understanding of this important issue and to offer insights into the potential of a new therapeutic option, ceragenins, in the context of their use in the form of core-shell nanosystems.

## 2. HAIs Caused by Multidrug-Resistant Gram-Negative Rods: Where We Are and Where We Could Go with New Treatment Strategies

According to the commissioned report by the UK Government about antimicrobial resistance, it has been posited that by the year 2050, antimicrobial resistance (AMR) has the potential to result in the loss of around 10 million lives annually [28,29]. Despite criticism of these forecasts, the World Health Organization (WHO) and several other societies and researchers agree that antimicrobial resistance (AMR) is a serious issue that requires a global action plan [30].

The resistance to antibiotics can be categorized as either innate, adaptive, or acquired by the organism [3]. Certain bacteria possess inherent resistance to specific antibiotics as a result of their unique cell wall composition, the functionality of efflux pumps, or the presence of porins [31]. Adaptive and acquired mechanisms may include (i) reduced drug penetration into the pathogen’s cell, (ii) drug removal from the bacterial cell, (iii) modification of drug targets through mutation selection, or (iv) rendering drugs inactive through enzymatic means [31]. The innate resistance means that all strains belonging to the same species exhibit insensitivity to a specific antibiotic. In contrast, acquired resistance occurs when certain strains within a given species develop resistance to an antibiotic that they were previously susceptible to. Mechanisms encompass two main processes: the occurrence of mutations in preexisting genes, such as those found in intracellular targets [32] or core metabolic genes [33], and the acquisition of novel antibiotic resistance genes through horizontal gene transfer (HGT) [31]. The latter facilitates the transmission of antimicrobial resistance genes within and between species, leading to the emergence of a pandemic of antimicrobial resistance [34,35]. Whatever the reason, antibiotic resistance already poses a severe threat to human health by making it more difficult to effectively treat infections [4]. Multidrug-resistant pathogens are causally linked to significant morbidity and death rates in HAIs owing to their acquired resistance to a wide range of medicines, including critical antibiotics like carbapenems and colistin [36]. Within this group of pathogens, approximately 16% to 20% exhibit multidrug-resistant (MDR) phenotypes. These phenotypes include methicillin-resistant *S. aureus* (MRSA), vancomycin-resistant *Enterococcus faecium* (VRE), cephalosporin-resistant *K. pneumoniae*, *E. coli*, and *Enterobacter* spp. as well as Gram-negative rods (*K. pneumoniae*, *Escherichia coli*, *Enterobacter* spp., *P. aeruginosa*, and *A. baumannii)* resistant to most β-lactam antibiotics; even carbapenems [37,38,39].

The global rise in drug-resistant bacterial infections has led to a growing demand for novel antibacterial therapies [40]. This issue has garnered significant global attention, resulting in various financing, regulatory, and legislative efforts aimed at revitalizing research and development in the field of antibacterial treatments. The 2022 data reveals a positive trend with respect to the augmented quantity of early-stage clinical candidates as compared to 2019. However, it is disheartening to note that the number of initial therapeutic approvals between 2020 and 2022 remained notably deficient. The monitoring of the transition of Phase-I and -II candidates into Phase-III and subsequent stages in the coming years will be of great importance [41]. Additionally, there was an increased prevalence of newly developed antibacterial pharmacophores in the initial stages of clinical trials. Out of the 26 candidates in phase I, a minimum of 18 were specifically designed to address infections caused by Gram-negative bacteria [42]. Plazomicin, eravacycline, temocillin, cefiderocol, ceftazidime/avibactam, ceftolozane/tazobactam, imipenem/relebactam, and meropenem/vaborbactam are among the antibiotics with significant efficacy against Gram-negative bacteria newly approved by the US Food and Drug Administration (FDA) and the European Medical Agency (EMA) [43]. The practice of combination therapy has become increasingly prevalent as a means to augment the effectiveness of current antibiotics and surmount resistance mechanisms. Recent research has investigated the efficacy of combining colistin with novel drugs or two antibiotics with distinct modes of action, revealing enhanced clinical outcomes in the treatment of multidrug-resistant Gram-negative infections [44,45]. Employing bacteriophage therapy has resurfaced as a promising approach for addressing multidrug-resistant (MDR) Gram-negative infections. Current research focuses on isolating and characterizing bacteriophages that target specific bacteria strains, providing a personalized therapeutic approach [46]. Immunotherapies have been created, such as monoclonal antibodies that specifically target outer membrane proteins and virulence factors of multidrug-resistant Gram-negative bacteria. The antibodies have the ability to augment the immune response of the host and counteract the harmful effects of bacterial toxins. Additionally, ongoing research is focused on the creation of vaccinations that specifically target multidrug-resistant Gram-negative bacteria, with the goal of preventing infections and decreasing reliance on antibiotics. Furthermore, there has been a notable rise in the quantity of “nontraditional antimicrobials” [47,48] that are currently undergoing active evaluation in clinical studies [48]. Non-conventional antibacterial agents encompass a range of entities, including small molecules, monoclonal antibodies (mAbs), proteins, and live biotherapeutics such as bacteria and bacteriophages [42]. These agents primarily exert their effects on bacterial growth or virulence indirectly, employing diverse mechanisms such as toxin binding, reduction of cell adherence, inhibition of antivirulence targets, and modification of drug resistance [48]. Despite the positive developments in phase -I and -II antibacterial drug candidates, the proposed strategies to tackle late-stage pipeline challenges, and the current portfolio of licensed antibacterial agents, when combined with conventional medicines in late-stage development, antibacterial drug discovery and development must maintain a sense of urgency.

Antimicrobial peptides (AMPs) present a potentially compelling and attractive means for antibiotics. The AMP Colistin (Polymyxin E), derived from *Paenibacillus polymyxa* var. *colistinus*, is currently employed as an initial treatment option for multidrug-resistant *Acinetobacter baumannii* (MDR-AB) [49]. However, its usage is constrained due to the adverse effects of nephrotoxicity and the escalating prevalence of resistance [50]. Previous studies have documented the efficacy of several peptides (such as LL-37, a human antimicrobial peptide, and WAM-1, a marsupial AMP) against some pathogens in animal models of infection [51]. The findings revealed that both peptides, LL-37 and WARM-1, exhibited the ability to suppress biofilm formation in all clinical isolates at certain concentrations. Additionally, WAM-1 was seen to disperse mature biofilm in the majority of isolates [51]. While the antibacterial properties of LL-37 are weakened when exposed to human serum, this phenomenon does not occur in the case of WAM-1. There is a requirement for further new drugs that can effectively target unmet therapeutic requirements. These molecules must evade established pathways of medication resistance and align with contemporary reimbursement procedures.

## 3. Physicochemical Properties of Ceragenins and Ceragenin-Based Nanosystems That Defined Their Antibacterial Properties and Specified Bacterial Targeting

**Figure 1:**
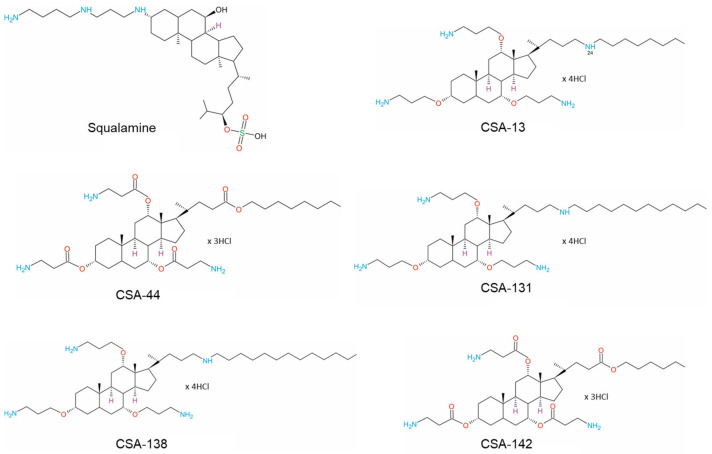
Structural formulae of squalamine and ceragenins CSA-13, CSA-44, CSA-131, CSA-138, and CSA-142.

Ceragenins, small-molecule mimics of endogenous antimicrobial peptides (AMPs), naturally produced by the immune system in all mammals, have attracted considerable interest due to their potent antimicrobial properties [52,53]. The first discovered natural molecule shearing characteristic of synthetic ceragenins is squalamine, which is naturally present in the stomach of *Squalus acanthias* sharks. In the preliminary phases of the investigation involving ceragenins, scientists created a variety of novel agents that increase the susceptibility of Gram-negative bacteria. This was accomplished by considering the bactericidal properties of polymyxin B and its metabolites. They synthesized numerous cholic acid derivatives, including derivatives with amine and guanidine functionality. In addition to possessing antibacterial properties against Gram-negative and Gram-positive bacteria, the researchers discovered that these compounds inhibited the biofilm formation of bacteria. The aforementioned class of cholic acid derivatives became known as cationic steroid antimicrobials (CSAs) or ceragenins over time (Figure 1).

Simultaneously, it has been observed that CSAs exhibit a low level of toxicity, providing evidence to support the potential clinical application of these compounds [54]. Numerous generations of CSAs have been synthesized and subjected to comprehensive evaluation. The compounds indicated as CSA-1 to CSA-50 have been categorized as constituents of the first generation of CSAs. While the remaining compounds synthesized so far are part of the second generation of CSAs [55].

**Figure 2:**
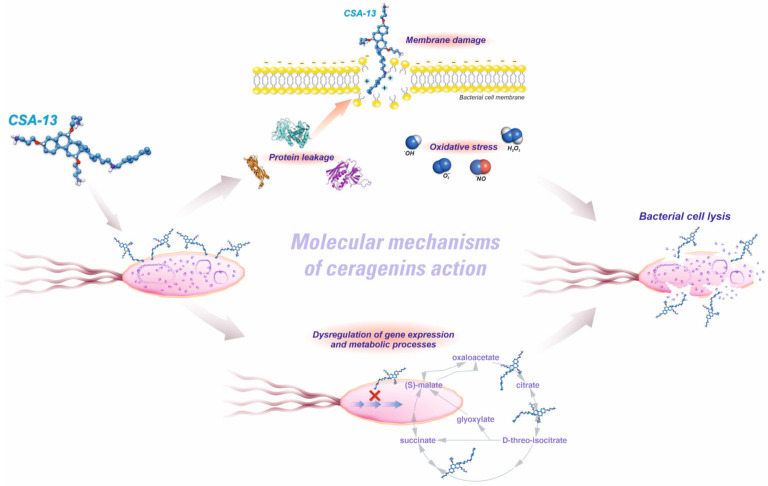
Molecular mechanisms of ceragenin action.

The cationic nature of ceragenins is considered to be one of their defining characteristics, which enables them to engage in electrostatic interactions with microbial membrane components that possess a negative charge [56]. The positive charge of these entities arises from the inclusion of amino groups in their structural composition. This interaction promotes the adsorption of ceragenins onto the membranes and subsequently leads to their membrane insertion, which leads to the rupture of the membranes (Figure 2).

The membrane-disrupting activity of ceragenins is dependent on the presence of hydrophobic parts, which integrate into the lipid bilayer of microbial membranes. In contrast to traditional antibiotics, which selectively act on specific cellular processes or proteins, ceragenins possess a membrane-disrupting effect that poses a significant obstacle for bacteria to acquire resistance [57]. This is particularly relevant when addressing multidrug-resistant bacteria that have undergone the development of numerous resistance mechanisms. A recent study presented the hypothesis that ceragenins have a distinctive mode of action, and scientists proposed a model in which ceragenins cross the outer layers of the bacterial envelope and target, more specifically, the inner membrane [58]. Moreover, the publication presented by Durnaś et al. [59] shows that ceragenins, due to their structural similarity to natural antimicrobial peptides, also have a similar mechanism of action, specifically to induce damage and impairment of the plasma membrane, leading to fungal cell malfunction. Furthermore, the utilization of scanning electron microscopy (SEM) and atomic force microscopy (AFM) has revealed that CSAs exhibit a propensity for interacting with plasma membranes, as evidenced by the presence of distorted cell surfaces and significant alterations in the morphology of Candida cells. Furthermore, another study has demonstrated that the primary mode of fungicidal activity exhibited by ceragenin is attributed to the generation of reactive oxygen species (ROS) [60]. This process results in the deterioration of the fungal membrane and the subsequent release of internal components, finally leading to the demise of the fungal organism. In addition, the amphiphilicity of ceragenins is achieved through precise engineering of the hydrophilic and hydrophobic areas, thereby establishing a delicate balance. The maintenance of this equilibrium is of utmost importance for their capacity to engage with the hydrophilic surface of microbial membranes as well as infiltrate the hydrophobic core, ultimately resulting in the rupture of the membrane. Position C-24 holds significant importance as it is closely associated with the groups that have a large impact on the bactericidal activity of CSAs [61]. During this study investigating cationic steroid antibiotics, it was observed that compounds with longer hydrophobic chains at C-24 exhibit potent bactericidal activity against both Gram-negative and Gram-positive bacteria, while those with shorter chains are less effective against Gram-negative strains while retaining strong activity against Gram-positive organisms. To improve cell selectivity, a polyamine was attached at C-24, reducing hemolytic activity while maintaining antibacterial efficacy. Amino acids were also introduced to facilitate specific binding interactions, potentially increasing affinity and selectivity. The justification for employing amino acids is the ability of the amine groups to offer cationic recognition, while the side chains may contribute further associative contacts with a particular binding target. Consequently, this enhances the affinity and, potentially, the selectivity of the interaction. The aforementioned correlations represent fundamental observations; however, ongoing research is being conducted to enhance the precision of this set of molecules.

Ceragenins, being non-peptide-based compounds, do not serve as substrates for proteases, resulting in their exceptional stability under physiological conditions. These compounds are not susceptible to proteolysis and are inexpensive to manufacture, making them an attractive alternative to peptide-based synthetic CAMPs [62]. One of the primary benefits of ceragenins is their resistance to the development of microbial resistance and their ability to target the bacterial cell membrane [63,64]. Traditional antibiotics frequently encounter the problem of bacteria evolving resistance mechanisms, reducing their effectiveness over time.

The research conducted by Li, Chunhong, et al. demonstrated that the effectiveness of ceragenins in combating Gram-negative bacteria is influenced, at least in part, by the length of the lipid chain attached to the molecule [65]. The lipid chain was postulated to serve as a means of anchoring the bacteria to its outer membrane. It has been proven that CSA-131, containing a lipid chain consisting of 12 carbon atoms, exhibited superior activity compared to CSA-13 and CSA-44, which both possess lipid chains consisting of eight carbon atoms, in contrast to CSA-142, which possesses a six-carbon chain and exhibited the lowest level of activity [66,67,68,69].

**Figure 3:**
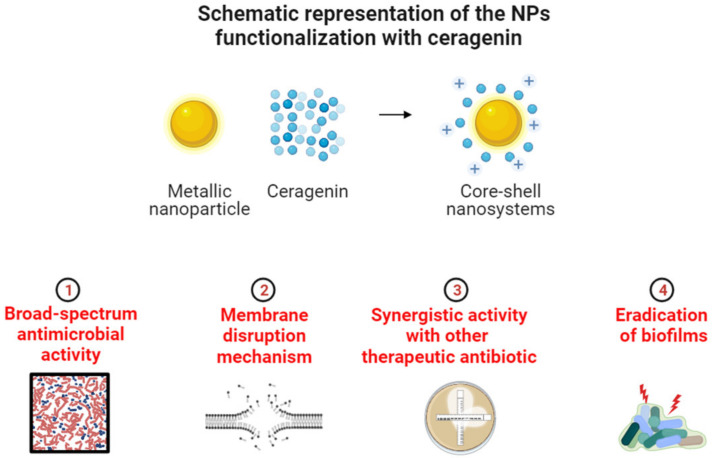
Therapeutic strategies for the treatment of the gram-negative rods by ceragenins and ceragenin-based core-shell nanosystems.

The incorporation of ceragenins into nanosystems improves their antibacterial properties and targeting abilities. Nanostructures can encapsulate and deliver ceragenins to infection sites, thereby enhancing their local concentration and efficacy while decreasing off-target effects. Physicochemical characteristics of ceragenin-loaded nanosystems, such as size and surface charge, play a crucial role in bacterial targeting. Due to their enhanced permeability and retention effect, nanoparticles within the optimal size range can promote passive accumulation within bacterial biofilms. Surface charge manipulation enables controlled interactions with bacteria and can increase specificity by targeting the surface properties of bacteria. Utilizing the synergistic benefits of both ceragenins and nanostructures, ceragenin-based nanosystems have emerged as an attractive approach to combating bacterial infections (Figure 3).

The presence of magnetic nanoparticles leads to enhanced incorporation and/or absorption of membrane-active substances like ceragenins and conventional antibiotics such as colistin or vancomycin into bacterial cell wall compounds. This interaction results in membrane disruption, leading to the release of intracellular contents as well as the induction of oxidative stress by the magnetic nanoparticles. Consequently, the organelles within bacterial cells experience detrimental effects. Furthermore, the combination of metallic nanoparticles and ceragenins exhibits synergistic properties in combating bacteria in both planktonic and biofilm states, including multidrug-resistant strains. This promising development has potential for addressing the urgent worldwide challenge of growing microbial resistance.

The investigation and advancement of ceragenins’ and ceragenin-based core-shell nanosystems’ pharmacokinetics and toxicity are currently subjects of intensive scholarly inquiry and continuous progress. The distinctive attributes of these entities provide both benefits and constraints, prompting continuous endeavors to optimize their properties to enhance their therapeutic potential. The study conducted by Wnorowska et al. [70] demonstrated that CSA-13 exhibits bladder accumulation within the initial 4-h period, with subsequent detection in the liver after 8 h. Following a 24 -h period, CSAs were completely eliminated. This finding aligns with prior research indicating the renal accumulation of ceragenin, hence reinforcing the credibility of ceragenin’s application in treating urinary tract infections [71]. The veracity of these findings was substantiated through an examination of animal excrement. The investigation of the impact of urinary pH on the pharmacokinetic and antibacterial properties of ceragenins is of great importance, considering the significance of urine ceragenin concentrations in the treatment of urinary tract infections and the involvement of kidneys in CSA-13 clearance. Moreover, in another in vivo study aiming to elucidate the anti-cancer potential of CSA-containing nanosystems, the compound AuP@CSA-131 exhibited distinct characteristics such as delayed clearance and extended blood circulation in comparison to free ceragenin, with subsequent improvement of biological activities [72].

## 4. Activity of Ceragenins and Ceragenin-Based Nanosystems against Multi-Drug Resistance Gram-Negative Rods

One of the major challenges encountered in combating multidrug resistance pertains to the high occurrence of pathogens such as *Pseudomonas aeruginosa* (*P. aeruginosa*), *Acinetobacter baumannii* (*A. baumannii*), *Klebsiella pneumoniae* (*K. pneumoniae*), and *Enterobacter* spp. [33]. These pathogens are primarily responsible for nosocomial infections, underscoring the pressing need for novel and effective therapeutic strategies [73] and are the focus of this review.

### 4.1. Ceragenins as Potent Antimicrobials with a Broad-Spectrum of Activity

Multiple investigations, including our own, have provided a growing body of evidence of the efficacy of ceragenins and ceragenin-based nanosystems in combating multidrug-resistant strains. The assessment of the antibacterial properties of these drugs is commonly conducted by determining the minimum inhibitory concentrations (MICs) and minimum bactericidal concentrations (MBCs).

To date, a number of studies report promising results on the utilization of ceragenins to combat HAIs. The findings of one of the investigations indicate that a selection of ceragenins (CSA-13, CSA-44, CSA-131, CSA-138, and CSA-142) exhibit efficacy against 50 multidrug-resistant strains of *K. pneumoniae* displaying a high level of resistance to colistin [66]. Another study showed that CSA-13 exhibited antimicrobial properties within a range of 2-6 mg/L, while CSA-142 demonstrated similar effects [74]. Furthermore, the minimum inhibitory concentration values for meropenem-resistant clinical isolates of *Klebsiella pneumoniae* were seen to range from 0.5 to 32 mg/L for CSA-13 and CSA-44, with the lowest concentration of CSAs that is effective in inhibiting the growth of 90% (MIC_90_) of *K. pneumoniae* being 32 mg/L [66]. Similarly, CSA-131 exhibited MIC values ranging from 0.5 to 16 mg/L, with a MIC_90_ of 32 mg/L. These findings were corroborated by another publication [67].

Importantly, ceragenin has also been proven to have strong effects against other multidrug-resistant strains, such as *P. aeruginosa* and *A. baumannii*. Carbapenems hold significant importance as commonly employed antipseudomonal medications, notwithstanding the escalating global prevalence of carbapenem resistance in *P. aeruginosa* strains. One of the most crucial categories to consider is carbapenem-resistant *P. aeruginosa* (CRPA) [75]. In instances of this nature, colistin is the sole available therapy choice. Nevertheless, the emergence of colistin resistance is being observed worldwide [76]. The presence of carbapenemases in CRPA strains has significant therapeutic implications due to their ability to confer resistance not only to carbapenems but also to other β-lactam antibiotics, including certain new β-lactam-β-lactamase inhibitors [77]. A potential correlation between the broader range of resistance and unfavorable outcomes in patients with CRPA infections may exist. The published data clearly show that the ranges of minimum inhibitory concentration values for ceragenins against 150 strains of *P. aeruginosa*, both with and without resistance mechanisms to β-lactams, are consistent across groups and comparable to the reference strain of *P. aeruginosa* ATCC 27853 [78]. The findings presented in the published study align with the results reported by Vila-Farrés et al. [79], who conducted experiments to evaluate the efficacy of several ceragenins, such as CSA-44, CSA-131, and CSA-138, against clinical isolates of *A. baumannii* and *P. aeruginosa*, including those that were resistant to colistin. In both studies, CSA-131 had the highest level of activity. The minimum inhibitory concentration values for the complete set of strains were determined to be 1 mg/L and 2 mg/L for the MIC50 and MIC90, respectively [79]. Similarly, a separate investigation examining the in vitro impact of ceragenins on 20 clinical strains of CRPA demonstrated that CSA-131 exhibited the highest level of activity among the ceragenins tested. Importantly, the antibacterial activity of ceragenins remains consistently effective regardless of the mechanisms of resistance observed in conventional antibiotics [69]. Moreover, the strong antibacterial effect of CSA-13 against *P. aeruginosa* as well as uropathogenic *E. coli* strains was also demonstrated in an in vivo model [70,71] (Table 2).

Interesting, in one of the more recent studies, Paprocka et al. [78] conducted a study to examine the activity of ceragenins CSA-13, CSA-131, and CSA-44 in comparison to three newly developed conventional beta-lactam antibiotics: (1) ceftolozane/tazobactam, (2) ceftazidime/avibactam (which have been available for several years since their approval in the US and Europe), and meropenem/vaborbactam (which was introduced into the medical market several years thereafter) against clinical strains of *P. aeruginosa* exhibiting various resistance patterns. The findings demonstrate the favorable efficacy of ceragenins against *P. aeruginosa*, including those that are susceptible to conventional antibiotics, as well as multidrug-resistant and extensively drug-resistant strains. Notably, the compounds exhibited activity against strains that manufacture metallo-beta-lactamases while retaining susceptibility solely to colistin. In comparison to traditional antibiotics, the minimum inhibitory concentration values for each ceragenin against *P. aeruginosa* strains, both with and without resistance mechanisms to β-lactams, exhibit similar ranges between the two groups. Furthermore, the antifungal activity of the ceragenins (CSA-13, CSA-131, and CSA-192) was compared to the impact of omiganan (the completion of phase 3 clinical trials evaluating the efficacy of omiganan 1% gel in preventing catheter-related bloodstream infections and providing topical skin antisepsis in healthy adult participants has been reported in the database of the U.S. National Institutes of Health) [59]. Ceragenins exhibit significantly greater candidacidal action in comparison to omiganan.

Among the several categories of nanoparticles, gold nanoparticles (Au NPs) have been recognized as highly beneficial materials in the field of nanomedicine [81]. It is important to emphasize that gold nanoparticles have garnered considerable interest due to their distinctive characteristics, such as their quite small size, easy surface modification, and low toxicity [82]. It is worth noting that a considerable number of gold nanoparticles have been employed as antibacterial agents in conjunction with small-molecule antibiotics, antimicrobial peptides, or cationic ligands attached to their surface [83]. The bactericidal properties of nanosystems consisting of ceragenin attached to the surface of nanoparticles in three different shapes: rods (AuR NPs@CSA-131), peanuts (AuP NPs@CSA-131), and stars (AuS NPs@CSA-131), have been evaluated against bacterial strains with various mechanisms of resistance to antibiotics (e.g., *K. pneumoniae* ESBL+, *K. oxytoca* ESBL+, *P. aeruginosa* LESB58 highly resistant to antibiotics with production of chromosomally encoded inducible AmpC-β-lactamase, and *P. aeruginosa* with the active efflux pumps) [84]. Significantly, the enhanced efficacy of the nanosystem in comparison to ceragenin arises primarily from the localized augmentation of ceragenin concentration and its enhanced internalization by cells, rather than from the inherent antibacterial properties of nanogold. While our previous research has shown that gold nanoparticles with varying shapes may possess antimicrobial [85] and anti-cancer properties [86], it is important to note that achieving these effects typically requires higher doses of nanoparticles compared to those found in the nanosystems currently being developed.

In another report, we demonstrated that improvement of the antimicrobial activity of CSA-13 is also possible by immobilizing CSA onto the surface of iron oxide-based magnetic nanoparticles. As demonstrated, nanocompounds, referred to as MNP-CSA-13, significantly augmented the antibacterial efficacy against multidrug-resistant *P. aeruginosa* [87]. Moreover, such nanoformulation enhances the compounds’ hemocompatibility as a result [88,89].

### 4.2. Enhancing Antibiotic Potency: Unveiling the Synergistic Activity of Ceragenins with Conventional Antibiotics

To address the issue of resistance strains, a potential strategy involves the utilization of a combination of different antibiotics as a countermeasure. This method can increase the pathogen target spectrum and reduce antibiotic use in therapeutic settings, reducing drug resistance. Moreover, the implementation of combination therapy has the potential to mitigate toxicity concerns by enabling the administration of reduced dosages of the respective deleterious medications in question. The effective application of synergism as a strategic approach involves the combination of many therapeutic agents with the aim of achieving a therapy that is more efficacious while also mitigating the rapid development of resistance.

Some of the first work evaluating the synergistic effect of ceragenin with conventional antibiotics inspired the evaluation of this effect against multidrug-resistant strains [90]. In 2008, the activity of CSA-13 against clinical isolates of *P. aeruginosa*, including MDR-*P. aeruginosa*, was evaluated. It has been proven that CSA-13 shows synergy with cefepime and ciprofloxacin against *P. aeruginosa* [90]. The above results were confirmed in another study that indicates a synergistic effect for CSA-13 in combination with colistin against clinical strains of *P. aeruginosa* isolated from cystic fibrosis patients (synergism was observed for 54% of tested strains) [91].

*A. baumannii* strains that were resistant to carbapenems were tested in vitro using CSA-13 in combination with colistin, tobramycin, and ciprofloxacin [92]. The outcomes of this investigation showed that synergistic interactions were identified in all combinations. With the use of the CSA-13 and colistin combination, the bactericidal effect in 55% of the tested strains showed the most synergistic interactions, whereas with the CSA-13 and tobramycin combination, 35% of the tested strains showed the least synergistic interactions. Then, recently published data showed the in vitro activities of ceragenins (CSA-8, CSA-13, CSA-44, CSA-131, and CSA-138) in combination with colistin against 25 carbapenem-resistant *A. baumannii* strains [80]. In agreement with the previous reports, a synergistic effect of CSA-13 with colistin was confirmed. Moreover, combinations of CSA-13 with CSA-131, CSA-13 with CSA-138, and CSA-131 with CSA-138 were found to have a synergistic effect. Importantly, no evidence of an antagonistic effect was found.

Another study also examined the synergistic effects of two ceragenins with colistin or meropenem on six *K. pneumoniae* strains showing different resistance patterns. The results indicate that the MIC values of ceragenins were similar or better than the antibiotics tested, except for colistin, and the synergistic effect of CSA-131 in combination with colistin was found for the strains at both 1 × MIC and 4 × MIC [66].

These results are extremely promising in the fight against multidrug-resistant strains of Gram-negative bacteria in particular, due to the rapid development of resistance to various antibiotics.

### 4.3. Demonstrating the Proven Anti-Biofilm Efficacy of Ceragenin for Clinical Application

Numerous bacterial species possess the capacity to form biofilms, which consist of structured communities of bacteria that adhere to both biotic and abiotic surfaces, facilitated by the secretion of extracellular matrix [93]. Biofilms play a crucial role in facilitating bacterial resistance to antibiotics by providing a protective environment for bacteria within a matrix [94,95]. Within biofilms, bacterial cells exhibit significantly higher tolerance to antimicrobial agents, with tolerance levels reaching up to 10,000 times greater than those observed in planktonic cells [96].

Prior research has indicated that there is a significant increase in the prevalence of *K. pneumoniae* strains that exhibit resistance to a wide range of antibiotics, particularly when these bacteria possess the ability to build biofilms [97]. Treatment of infections caused by biofilm-forming *K. pneumoniae* strains is more difficult than for other strains [98]. Several research studies have demonstrated a correlation between the ability of bacteria to form biofilms, which are regulated by genes and proteins, and their resistance to colistin [99,100]. Studies have also depicted the interrelationships- between biofilm formation, gene/protein regulation, and the resistance phenotype [96]. Given that over 60% of infections are linked to the capacity of microorganisms to form biofilms, the substantial efficacy of ceragenins in inhibiting biofilm formation has been substantiated by numerous prior investigations [101]. An example of this is the study conducted by Chmielewska et al., which provided evidence of the efficacy of CSA-13, CSA-44, and CSA-131 in combating biofilm formation by NDM-1 (New Delhi metallo-β-lactamase-1)-generating strains of *Klebsiella pneumoniae* BAA-2472 and BAA-2473 [102]. The ranges of MIC and MIB values for ceragenins CSA-13, CSA-44, and CSA-131 against *K. pneumoniae* subsp. *pneumoniae* BAA-2472 and *K. pneumoniae* BAA-2473 were, respectively, 1 to 2 mg/L and 1 to 4 mg/L, and were several times lower compared to the majority of antibiotics used in this study (ertapenem, meropenem, gentamicin, tobramycin, ciprofloxacin, and fosfomycin). The lowest, identical values were obtained by ceragenins CSA-13 and CSA-131 [102]. In addition, data are available that show the activity of ceragenins CSA-13 and CSA-90 against both single- and multi-species biofilms formed by *P. aeruginosa*, *A. baumannii*, *E. coli*, *K. pneumoniae*, and *Candida albicans* [101]. Several studies have shown that ceragenins, especially CSA-13, have antibiofilm activity against *P. aeruginosa* [103,104,105]. Interestingly, *P. aeruginosa* biofilm usually produces a large amount of a Pf1-like virus compared with phage production in planktonic *P. aeruginosa* cultures [106]. In connection with this fact, we presented the results that demonstrated that the Pf1 bacteriophage dramatically reduces the bactericidal activity of the cathelicidin LL-37 peptide, but it has no effect on the ability of synthetic cationic lipids like CSA-13 and CSA-131 to eradicate bacteria. The potential of cationic lipids to prevent and treat *P. aeruginosa* infections, particularly those brought on by LES strains in CF airways, is suggested by their capacity to prevent *P. aeruginosa* LESB58 biofilm formation and eliminate *P. aeruginosa* LESB58 bacteria from CF sputum in vitro [107].

It is important to mention that the immobilization of CSA-13 on magnetic nanoparticles resulted in a significant enhancement of its bactericidal and anti-biofilm properties in PBS-based conditions. Additionally, the antimicrobial activity of CSA-13 was further intensified when exposed to bodily fluids, including urine, saliva, plasma, pus, ascites, cerebrospinal fluid, bronchoalveolar lavage, and cystic fibrosis sputum [87,108]. The data presented in this study emphasizes the therapeutic efficacy of MNPs, as evidenced by the enhanced bactericidal effects and biofilm prevention capabilities observed when combining CSA-13 and CSA-131 with MNPs against *P. aeruginosa* Xen 5 and other Gram-negative bacteria [108,109].

### 4.4. Investigating Morphological Alterations in Bacterial Cells following Exposure to Ceragenins

**Figure 4:**
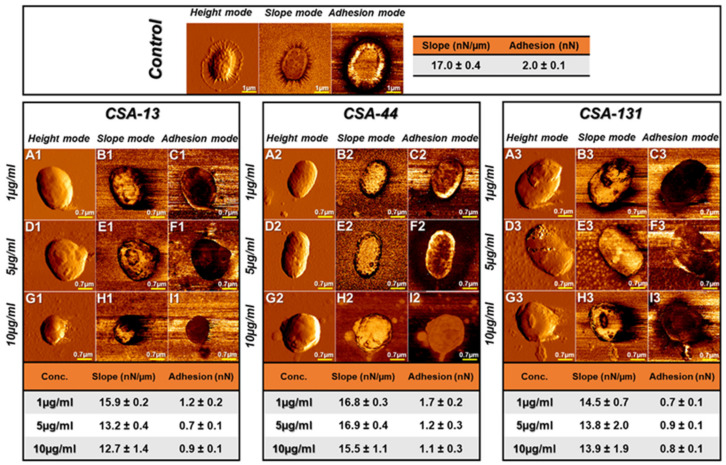
Morphological changes of *K. pneumoniae* in AFM before and after application of ceragenins CSA-13, CSA-44, and CSA-131 [67].

The direct observation of the changes in morphology of microorganisms in response to antibiotic exposure is one of the most often used techniques to assess bactericidal effects [110]. Frequently, atomic force microscopes (AFM) that permit observation of live microorganisms are used in this regard. Measurements of the physicochemical properties of bacterial cells, followed by changes in the architecture of the bacterial cell membrane caused by antibiotic exposure, can reveal important details about the putative mechanisms of action of the antimicrobial agents being studied. This technique was successfully employed to explore the morphological changes in *K. pneumoniae* BAA-2473 cells after exposure to ceragenins (CSA-13, CSA-44, and CSA-131) (Figure 4) [67]. 

The main objective was to gain a better understanding of the mechanisms by which ceragenins induce membrane damage. The findings of the investigation suggest that the method by which ceragenins exert their effects may entail their interaction with negatively charged compounds present in the bacterial membrane, such as lipopolysaccharide (LPS) and phosphatidylglycerol (PG). This form of interaction gives rise to the reorganization of membrane compound packing, ultimately resulting in the disruption of the membrane. Observed changes in AFM include changes in cell membrane morphology such as microcracks and surface wrinkles.

**Figure 5:**
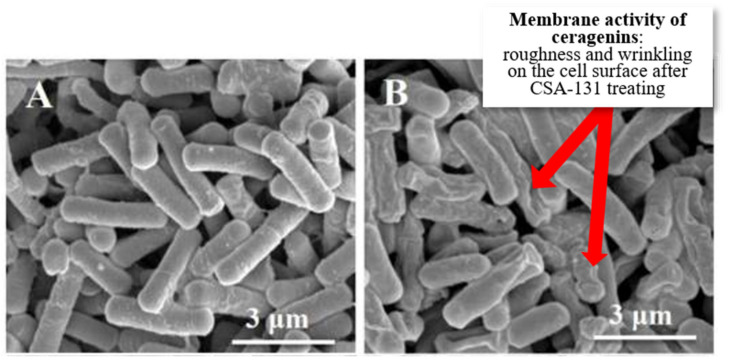
Changes in morphology in SEM after application of ceragenin CSA-131 against *K. pneumoniae* ((**A**)—before application of CSA-131, (**B**)—after application of CSA-131) [109].

A change in shape and a decrease in bacterial adhesion and stiffness were also observed. Changes in bacterial morphology can also be observed in the SEM (Figure 5). After applying ceragenins, the bacterial images show folding, wrinkling, and discontinuity of the membranes, which indicates membrane activity (insertion) of ceragenins [109].

Other published data presented changes in cell shape and mechanical characteristics of *P. aeruginosa* after treatment with MNP-CSA-13 in order to confirm the antibacterial activity of ceragenin-functionalized magnetic nanoparticles by atomic force microscopy (AFM) [110]. The findings show that nanosystems of MNP-CSA-13 attach to bacteria before rupturing the cell membrane, allowing the internal contents to escape. Interestingly, the addition of unfunctionalized MNPs causes bacterial aggregation [110].

## 5. Conclusions

In the ongoing effort to combat nosocomial infections, specifically those caused by multidrug-resistant strains of *K. pneumoniae*, *P. aeruginosa*, and *A. baumannii*, the introduction of ceragenins and ceragenin-based core-shell nanosystems as a viable alternative represents a significant advancement in infection management and future therapy. The diverse characteristics of ceragenins, such as their wide-ranging antibacterial properties, distinctive method of action in disrupting membranes, and proven effectiveness *in vivo*, all highlight their potential as a significant addition to our therapeutic arsenal. In our endeavor to combat multidrug-resistant bacteria, ceragenins offer a compelling opportunity, thanks to their remarkable effectiveness and resilience against the emergence of resistance. Through ongoing research, ceragenins have the potential to significantly transform the field of infection management.

## Figures and Tables

**Table 1:** Risk factors and etiological agents of health care-associated infections. The table presented is derived mainly from information extracted from the following publication [7].

Type of Nosocomial Infections	Pathogens	Risk Factors	References
Central Line-Associated Blood Stream Infection (CLABSI)	Coagulase-negative *Staphylococcus*, *Staphylococcus aureus*, *Enterococcus* spp., *Streptococcus* spp., *Candida* spp., *Acinetobacter baumannii*, *Escherichia coli*	Chronic illness, neutropenia, malnutrition, parenteral nutrition, extremes of ages, and bone marrow transplantations, prolonged hospitalization before catheterization, prolonged time of catheterization, multi-lumen CVC, type of catheter material, multiple CVC, urgent insertion, and lack of sterile barriers or breaks in the aseptic technique	[8,9,10,11,12]
Catheter-Associated Urinary Tract Infection (CAUTI)	*Escherichia coli*, *Klebsiella* spp., *Acinetobacter baumannii*, *Pseudomonas aeruginosa*, *Enterococcus* spp., and *Candida* spp.	Duration of catheterization, female sex, paraplegia, cerebrovascular disease, older age, diabetes mellitus, history of UTI in the preceding year, and recent antibiotic use within 90 days	[8,10,12,13,14,15]
Skin and Soft Tissue Infection (SSI)	*Staphylococcus aureus, Escherichia coli*, *Klebsiella* spp., *Enterobacter* spp., *Pseudomonas aeruginosa*, *Acinetobacter baumannii*, *Enterococcus* spp.	Duration of surgery, wound class, hypothermia and hypovolemia during surgery, hypoxemia, the urgency of surgery, more than one intervention/surgery, necessity for blood transfusion, and the type of prosthesis implanted, wound class, and duration of operation due to the time that the tissue is exposed to the environment, leading to an increased chance of contamination, immunosuppression, tobacco use, obesity, hyperglycemia, malnutrition, joint disease, and increasing age	[8,10,12,13,16,17]
Hospital-acquired pneumonia (HAP) and ventilator-associated pneumonia (VAP)	*Klebsiella pneumoniae*, *Enterobacter* spp., *Pseudomonas aeruginosa*, *Acinetobacter baumannii*, and *Staphylococcus aureus*	Prior IV antibiotic use within the last 90 days, need for ventilatory support, septic shock at the time of VAP, acute respiratory distress syndrome preceding VAP, more than five days of hospitalization before VAP onset, and need for acute renal replacement therapy	[18,19,20,21,22,23]
*Clostridioides difficile* Infection (CDI)	*Clostridioides difficile*	Antibiotic use and environmental contamination, both of which are modifiable risk factors. Other frequently seen risk factors include increasing age, hospitalization, multiple comorbidities, the use of gastric acid-suppressing medications, and immunosuppression	[8,24,25]

**Table 2:** Selected results summarize the values of the antibacterial activities of ceragenins and antibiotics against Gram-negative bacteria.

Tested Antimicrobial Agents	Minimal Inhibitory Concentration (mg/L)							
	*A. baumannii* Colistin-Resistant n = 1	*A. baumannii* Carbapenem-Resistant n = 25	*P. aeruginosa* Various Mechanisms of Resistance n = 150	*P. aeruginosa* Colistin-Resistant n = 1	*K. pneumoniae* Colistin-Resistant n = 5	*K. pnumoniae* MDR n = 50	*K. pneumoniae* NDM-1 n = 1	*K. pneumoniae* NDM-1 n = 1
CSA-13	4	1–16	0.5–8	<0.5	2–6	0.5–32	2	2
CSA-44	4	8–16	0.5–8	1	1–2	0.5–32	2	2
CSA-131	2	1–8	0.5–4	<0.5	1–3	0.5–16	2	2
CSA-138	4	4–32	-	1	2–8	1–32	-	-
Colistin	-	0.125–4	0.125–8	-	16–200	0.03–128	1	0.5
Meropenem	-	8–128	-	-	-	0.5–128	128	32
Reference	[79]	[80]	[78]	[79]	[74]	[66]	[67]	

## Data Availability

Not applicable.
